# Globetrotting Horses: Welfare Discourses and Disciplinary Power in the Transportation of Horses by Air

**DOI:** 10.3390/ani14131862

**Published:** 2024-06-24

**Authors:** Lucia Gräschke

**Affiliations:** Department of Geographical and Historical Studies, University of Eastern Finland, 80100 Joensuu, Finland; luciagra@uef.fi

**Keywords:** horse transportation by air, equine welfare, welfare discourses, disciplinary power

## Abstract

**Simple Summary:**

Every year, many horses are transported by air. Alongside sport horses traveling to tournaments worldwide, mainly breeding horses, such as shuttle stallions and broodmares, thoroughbreds traded at auctions, and leisure horses are transported by air. Although the transportation of horses by air is a well-established business with decades of experience, research has highlighted welfare concerns for horses and calls for the reform of international transportation standards. This study offers an overview of the existing equine welfare discourses and transportation practices within the business. Furthermore, we reveal how these well-established discourses and practices shape the behavior and identity of agency workers, aviation staff, and horses. The study’s conclusion can help improve the international agreements that set welfare standards for the transport of horses by air.

**Abstract:**

Every year, many horses are transported by air. Alongside sport horses traveling to tournaments worldwide, mainly breeding horses, such as shuttle stallions and broodmares, thoroughbreds traded at auctions, and leisure horses are transported by air. Research in veterinary science has highlighted welfare concerns during air transportation. Equine welfare is constituted in the language and discourse evolving from social, political, and ethical views about the treatment of horses. Consequently, this study targets power in creating equine welfare by analyzing the welfare discourses, transportation practices that generate welfare, and their impact on horses and humans in the transportation of horses by air. In detail, this research uses a Foucauldian discourse analysis to examine how welfare discourses and linked transportation practices constitute horses and humans using disciplinary power. The empirical material consists of 81 newspaper articles about horse transportation by air, five video clips, and four interviews with representatives of horse transport agencies that have set standards for the transportation of horses by air. The analysis discovers four different welfare discourses and various practices that guide the carrying of horses by air. The discourses have created inactive horses and human professionals in the business of horse transportation by air.

## 1. Introduction

More-than-humans, including equines, have a fundamental role in constituting and co-constituting not only the relations of humans but social relations in general [1]. Consequently, horses are embedded in modern ideas and practices about national identity, science and technology, breeding, trade, and commodification [2,3]. Unsurprisingly, globalization has increased not only the mobility of humans but also that of horses. Shuttle stallions travel around the globe, following summer and winter breeding schedules. Equestrianism, including disciplines like show jumping and horse racing, requires horses to participate in international tournaments [4,5]. Thoroughbred horses change owners at auctions with an international audience, and leisure horses relocate internationally with their owners or because of a change in ownership. The international transportation business includes travel agents for horses, airlines specialized in transporting horses, quarantine stations, horse facilities at airports, travel vets, and trained staff such as flying grooms. Furthermore, international guidelines, like the Live Animal Regulations and the Terrestrial Animal Health Code, guarantee equine welfare standards during transportation [6,7].

The horse and aviation industry has agreed that horses respond well to air transportation [8]. Studies in the field of veterinary sciences underpin the statement by measuring the stress levels, inflammation risks, and the acclimatization of horses during transportation [8,9,10,11,12]. Nevertheless, transport stress often occurs during loading, unloading, take-off, and landing [8]. Furthermore, recent research has highlighted that body weight loss, dehydration, leukocytosis, and respiratory and gastroenteric disorders have been associated with air transportation [13,14].

The existing welfare concerns highlight the various positions about welfare and introduce the role of power in constructing equine welfare practices in horse transportation by air. In other words, equine welfare is discursively constructed, changes over time, and is subject to power relations. Therefore, this study aims to identify welfare discourses that govern the debate about horse transportation by air, and their impact on humans and horses, with a Foucauldian discourse analysis. A Foucauldian discourse analysis explains the tensions between current welfare concerns when transporting horses by air, on the one hand, and a high degree of institutionalization of equine welfare through international agreements and agencies, on the other hand. In detail, the approach addresses how and why questions about welfare behaviors and beliefs in order to reveal generally accepted truths about equine welfare within the international transportation of horses by air. Instead of one universal truth, a Foucauldian discourse analysis is keen to expose common-sense truths, often appearing as taken-for-granted assumptions that have shaped individuals’ actions and beliefs [15]. Investigating the circulation of power through individuals thereby helps identify dominant, alternative, and counter-discourses. Thus, a Foucauldian discourse analysis depicts how welfare discourses and practices control humans and horses by self-regulating their behavior.

The guiding question of this research is as follows: How do discourses about equine welfare and linked transportation practices that generate equine welfare constitute horses and humans in the transportation of horses by air using disciplinary power? The theoretical framework to answer the question draws on Foucault’s concepts of disciplinary power [16], knowledge and discourse [17,18], and their applications to human–animal relations [19]. The empirical material encompasses newspaper articles, YouTube clips, and interviews with transport agency representatives specialized in horse transportation by air. The material has been analyzed using a Foucauldian multidimensional discourse analysis. In the following section, an overview of Foucault’s concepts of power to comprehend human–animal relations provides the theoretical foundation of this study.

### Equine Welfare and the Horse: Subject of Power and Object of Knowledge

Veterinarians, travel agents, and horse owners struggle with and for the concept of equine welfare because it has an ethical origin and not a purely scientific one. Consequently, Meijboom [20] noted that “science and ethics always go together” regarding animal welfare. While focusing on the moral positions towards animals is necessary to build an ethical frame concerning their treatment, science contributes empirical knowledge of animal welfare by analyzing their biology and behavior [20,21].

The term “animal welfare” comprises a variety of different views about the treatment of animals and their well-being, including but not limited to the dimensions of the animal’s pleasures and happiness, their freedoms, their ability to express natural behavior in a natural environment, and their capacity to flourish [22,23,24]. Therefore, we are following Meijboom [20] and comprehending equine welfare as a continuum that includes the plurality of moral and ethical positions about equine welfare. This continuum puts positions on the moral value of animals in relation to humanity’s ethical duties toward them. When animals only have instrumental value, there are no resulting duties towards them. When animals are morally considered as sentient beings, duties range from cruelty over discomfort to positive welfare. The view that animals have an intrinsic value is accompanied by another shift from positive welfare to the animal’s integrity. Lastly, the position that animals have inherent dignity demands a transition from respecting their integrity to respecting their autonomy [20]. In this view, instrumental value describes the use of horses for human ends, while intrinsic value stresses that horses have a value by themselves [25]. The recognition of horses as sentient beings underlines their ability to suffer.

The variety of different views turns equine welfare into an object of knowledge. In other words, equine welfare practices are negotiated and shaped in political, social, and economic spaces [26]. Michel Foucault’s contributions to discourse, knowledge, and disciplinary power provide a lens through which we can approach equine welfare and horse–human power relations in the transportation of horses by air. Although Foucault rejects the narrow definition of power, he carves out ways in which power operates in modern societies in a capillary manner through actors, institutions, and organizations. His work has been criticized as anthropocentric because of its focus on language and institutions, while having little interest in animals [27]. However, animal scholars have utilized Foucault to explore power in the relationships between humans and animals [19]. Palmer [27] distinguishes two starting points for approaching human and animal power relations from a Foucauldian perspective. Firstly, Foucault can be applied to uncover the construction of animals through discursive and cultural practices. In this view, animals remain objects of power and knowledge. Secondly, Foucault’s concepts of power can incorporate animals as agents and subjects to power by focusing on the interactions between humans and animals to reveal how animals and humans likewise experience and exercise power. By taking a post-humanist stance on power, this approach examines how power produces animal and human bodies [28,29]. Redmalm [28] provided an empirical study examining how pet-keeping practices simultaneously discipline pet keepers and pets. Włodarczyk [30] used Foucault’s biopolitics to illustrate historical changes in dog training by exploring how training methods discipline dogs and humans. This study also considers horses and humans together as subjects to power.

According to Foucault, power penetrates the body and produces subjects [16,31]. The subject has two meanings. Firstly, individuals become subject to someone else through control and dependence, and secondly, they become subject to their own identity through conscience and self-knowledge [32]. The second aspect is especially debated by animal studies scholars. The question of whether animals have a conscience or self-knowledge is itself the basis for concepts of animal agency and resistance to power. Carter and Charles [33] have identified two groups of responses among animal studies scholars as to whether animals have a sense of self. The first group argues that when animals have developed social forms and relations to a sophisticated extent, they likely have developed a sense of self. In this view, Palmer [27] noted that mammals and birds fall into the category of subjects, while many organisms and plants seem to remain excluded. The second group stresses that self-conscience is irrelevant to the existence of the animals’ agency. Actor–network theorists and Bruno Latour belong to this second group.

Foucault, too, was not primarily interested in the self-knowledge of subjects to tackle power [34]. Instead, he emphasized “the creative potential of power, generating truth, knowledge, societies and the living beings that populate them outside of the question of self-knowledge” [35]. Therefore, we comprehend that disciplinary power subtly works when producing a particular behavior. Concerning horses in the sport and entertainment industry, Hansen [36] showed that organizing and ordering space and time through training, cleaning, and feeding routines in specified spaces produces horses and their caregivers as subjects of power. In a similar way, we explore how welfare discourses and practices linked with these discourses produce horses and humans as subjects of power.

The production of subjects of power relies on objects of knowledge. Foucault [34] refers to the power/knowledge nexus. Here, power and knowledge are closely linked. Knowledge engenders power, and power needs knowledge to be exercised [34]. In the case of equine welfare during transportation, discourses organize and structure knowledge about the well-being of horses during air transportation by encompassing topics of transport logistics and organization, horse biology and behavior, and transport means and aviation. In the Foucauldian sense, discourse is not any piece of text but the meaning-making and ordering of things, objects, and practices through language [17]. In other words, discourse provides knowledge in a meaningful manner. Consequently, discourse is not just language but entails a material dimension because it produces practices that shape the objects of the discourse and hence subjects of power.

## 2. Empirical Data and Methodology

This study relies on three kinds of data to approach equine welfare as follows: newspaper articles about horse transportation by air published in openly accessible sources, videos published on YouTube about the topic, and interviews with representatives of horse transport agencies. To collect the data, we applied the following criteria: Firstly, a time frame of ten years (2012–2022) ensured a convenient overview of the newspaper articles and the YouTube clips. Secondly, we collected newspaper articles and YouTube clips published in English to gain empirical material designed for an international audience. Thirdly, we set a minimum of 5000 views for the YouTube clips and a minimum of the five highest-ranked clips. Both criteria indicate a high impact of the clips. Lastly, we did not set a fixed sample size for the interviews. Instead, we focused on the richness of the various perspectives reflecting the qualitative character of this study.

Consequently, we collected 81 English-speaking newspaper articles published worldwide between 2012 and 2022. The news articles were selected using a Google search in the news category using the keywords “horse transportation by air”. All news articles appearing in this search that focused on horse transportation by air were collected. The search was conducted in November 2022 and updated in January 2023 to include all relevant news articles from 2022. The articles are accessible online, and they were published in international newspapers, newspapers about international trade, magazines about horses and equine sports, and aviation magazines. Among others, the articles were published in the New York Times, Horse and Hound, Business Insider, Stat Times, and transportation and aviation magazines like FreightWaves and Air Cargo News.

Furthermore, we collected five video clips from YouTube with a basic search for “horse transportation by air”. All the clips selected appeared within the first six results, had more than 5000 views, were between one and nine minutes long, and were published between 2015 and 2021. One clip ranked among the first five clips had less than 5000 views and was not selected. Since a wide range of themes and information appeared within the first six clips, indicating thick or rich data of high quality [37], we limited the sample size to five clips. Two of the five clips were published by the International Federation for Equestrian Sports, an international organization that governs equestrian sports, one by the Equine International Air Freight, an Australian-based transport agency, one by World Horse Racing, an alliance of four iconic global racing festivals, and one by FedEx, an American logistics company that also ships horses.

Finally, we conducted four semi-structured online interviews with representatives of four horse travel agencies. Two agencies are located in Europe and two in North America. In detail, the interviewees occupied positions ranging from vet and proxy to manager. A total of four companies from 41 interview invitations responded positively to an interview request. Three interviews were conducted in English and one in German. Nevertheless, all participants emphasized their ability to participate in the interview using English if necessary. The interview records are between 30 and 60 minutes long and focus on the transportation of horses, covering three major topics—the procedure and organization of horse transport by air, the paperwork and certificates needed for horses to be internationally shipped, and animal behavior and welfare during transportation. The participants were particularly interested in sharing their stances and ideas about equine welfare during transportation by air. All interviews were recorded and transcribed, and informed consent was obtained. The different data sets provide an overview of public and professional voices within the business of horse transportation by air.

Foucault approaches discourse as “practices that systematically form objects of which they speak of” [17]. Although his work, The Archaeology of Knowledge [17], concerns discourse and language, Foucault never empirically tested the methodology and instead turned to analyze power [38]. Rather than a theory or a set of rules, a Foucauldian discourse analysis is a hermeneutic toolbox to investigate discourses. Using Foucault’s theory, we pay particular attention to discursive formation, which targets the ways in which meaning is produced through the different interpretations of words and vocabulary [17]. A Foucauldian discourse analysis focuses on a macro dimension of discourse [39] by considering systems, knowledge, ideology, and power. In contrast to micro- and meso-dimensions of discourse analysis that look at syntax, grammar, rhetorical tools, and the production of text in a particular environment, the macro dimension explores historical contexts and considers intertextuality to identify a hegemony of discourses [40,41].

A Foucauldian discourse analysis has broadly three dimensions—a historical inquiry, an investigation of mechanisms of powers, and an analysis of practices in which subjects are made up [42]. This analysis concentrates primarily on the second and third dimension. We used the coding software Atlas.ti 23 to create coding groups, categories, and labels that organized the reappearing themes, keywords, and practices concerning equine welfare. Furthermore, we coded the interviews, newspaper articles, and clips separately and subsequently compared their themes, keywords, and practices. For the video material, the study used a multimodal discourse analysis by examining sequences of language, music, and the visual content as a whole [43,44]. The following steps used to approach the data draw on Carabine [45], who provided a structured approach to Foucauldian discourse analysis.

Firstly, the content of the data was assessed to identify common themes and keywords around equine welfare. In detail, we searched for direct and indirect expressions and statements about equine welfare and practices indicating equine welfare. On their basis, we created coding groups and used labels that further specified the content. Secondly, the content was analyzed more closely. We identified reappearing welfare practices and themes. In addition, the interplay between practices and themes was examined to draw conclusions about the meaning-making of equine welfare. On the one hand, the practices and their explanations produce meaning for equine welfare themes. On the other hand, welfare themes give meaning to practices and highlight their relevance to equine welfare. Thirdly, we examined the relationship between the different themes and practices by checking if certain practices and themes appeared in clusters or opposed each other. Fourthly, we pooled the coding groups and summarized four categories constituting four major welfare discourses. Finally, we investigated how humans and horses were portrayed within the four categories. The representations illustrate how the discourses and their practices shape the behavior of humans and horses.

## 3. Discourses of Equine Welfare and the Construction of Professional Experts and Inactive Horses

### 3.1. Welfare as Providing Species-Appropriate Transport Conditions

**Figure 1:**
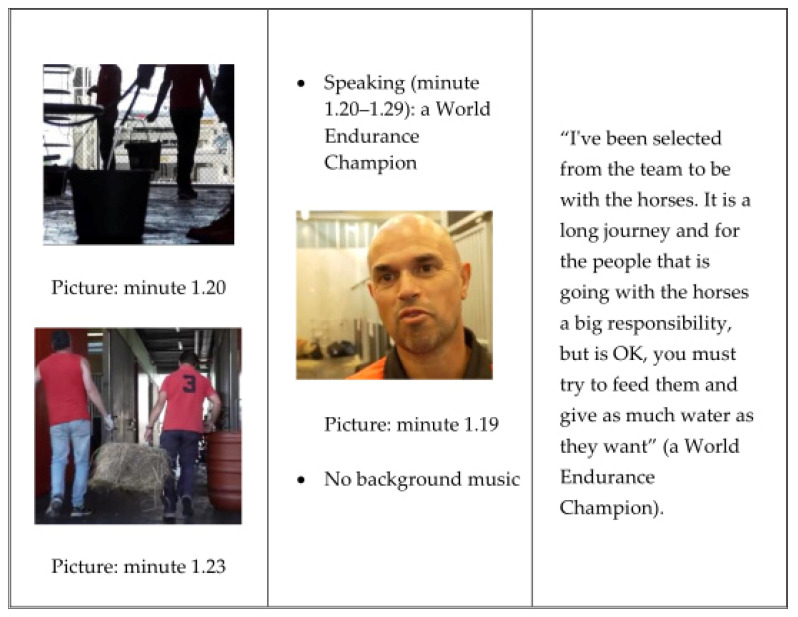
FEI 15.07.2021 [46].

The first welfare discourse targets equine needs and focuses on establishing horse-friendly transport conditions. In detail, equine welfare means the provision of a transport environment that answers equine-specific needs and preferences, such as providing feed and water, temperature regulation and ventilation, and the separation of male and female horses inside the aircraft. A sequence taken from a video clip illustrates the preparations for a flight, highlighting the provision of water and hay as a welfare practice (see Figure 1 [46]:

The sequence demonstrates the importance of hay and water for equine welfare during air transportation. The newspaper articles also frequently mentioned other feeds, like apple or carrot sticks, as snacks. An agency representative had noted that some horses do not drink much during transportation. As a result, grooms are instructed to provide water during and after the flight. The representative also recommended not hanging hay nets directly in front of the horses’ heads but lower. Otherwise, the animals might breathe in dust and particles of hay for the duration of the flight. Inhaling these particles can irritate the respiratory tract and increase the risk of developing shipping fever. In general, poor air circulation and dry air over a long period of time are associated with equine pleuropneumonia during transportation [47]. Furthermore, the newspaper articles and video clips included statements about temperature and ventilation inside the aircraft. As air stables store heat, the average temperature is roughly 17 °C inside the aircraft. This temperature is located towards the top end of the preferred temperature range of 5 to 25 °C for horses to maintain an average body temperature of between 37.5 and 38.5 °C, making the aircraft environment warm but not hot for horses [48]. Lastly, arranging horses by sex inside the aircraft adds to equine welfare. An agency representative summarized as follows:

“Very, very important is the load organization of the aircraft. […] For example, you must always put stallions in the front of the aircraft. So, they don’t have that much distraction. We spend an awful amount of time preparing load plans to ensure we have happy horses inside the airplane. We don’t necessarily know the horses, but you know the sex of the horses. And through experience, we can anticipate (agency representative 2).”

Loading plans minimize additional stressors for horses and make their handling easier for humans. Stallions and colts are male horses and get excited in the presence of mares. Female horses like mares and fillies, however, might feel stressed by their presence. As a result, stallions and colts are loaded in the front of the aircraft to reduce the visual and olfactory cognition of mares and fillies, who are loaded in the back. Geldings are often loaded in the middle of the aircraft. Lastly, two news articles mentioned that horses are not loaded together with perishables on the same cargo flight to avoid distraction.

This discourse is composed of the keywords, “horse”, “temperature”, “water”, “hay”, “sex of the horses”, “air circulation”, and “loading plans”. From this perspective, the discourse reflects three of the five freedoms of animals developed in the 1960s by the Brambell committee to promote animal welfare for domestic animals whose environment is primarily controlled by human activities [49]. In particular, the discourse highlights the freedom from hunger and thirst by providing feed and water, the freedom from discomfort by creating an appropriate environment using temperature and ventilation, and the freedom from fear and distress by separating horses by sex and from perishables. The five freedoms were developed for farm animals as a framework to set the minimum requirements to guarantee the elimination of negative welfare [49,50]. As a result, the discourse about species-appropriate transport conditions focuses on avoiding discomfort but sees animals as sentient beings regarding the continuum of welfare views [20].

Within the discourse, horses appear as passive members of a species, not individuals. Human activities dominate the welfare discourse by providing feed or water, making loading plans, and controlling the temperature inside the plane. Lastly, the quotations stress that knowledge about species in terms of biology has set the foundation for welfare practices. Such species-specific knowledge represents the scientific and cultural knowledge about horses. Foucault [16] refers to the double meaning of the word “discipline”. Discipline can refer to the scientific discipline of biology and the control of horses to exercise obedience. Disciplinary power circulating through bodies draws on the knowledge about equine behavior and biology present in the discourse. In other words, the creation of horse-specific transport conditions, like a relatively comfortable and ventilated environment, the separation of sexes, and the provision of feed and water, disciplines the horse bodies to remain inactive during transportation.

Following Foucault, discipline means making horse and human bodies docile and more useful, as docile bodies can be subjected, used, transformed, and improved [51]. Calm and inactive horses ensure unimpeded transportation. From the perspective of this welfare discourse, horses behaving calmly and inactively are healthy and happy horses. The disciplinary practices help horses relax and improve their well-being. The empirical material mentions that horses often doze or sleep during the flight. Furthermore, these environmental conditions discipline agency staff, flying grooms, and pilots, since agency workers spend time creating loading plans, while flying grooms arrange hay nets, provide water, and check on the horses during the flight, and pilots ensure the correct temperature inside the aircraft hold.

Schwan and Shapiro [51] point out that discipline enables economic control and provides techniques, like surveillance, that control laborers to increase productivity. Foucault draws a connection between discipline and capitalism. The discourse controls the activities of agency workers, flying grooms, and pilots by setting examples and standards for their work performance. Adapting to the horses’ needs creates expertise in agency workers, flying grooms, and pilots, making them professionals when transporting horses. Their labor is guided by welfare practices, enabling the improvement and monitoring of their work. In other words, performing these welfare practices shapes their expertise as professionals working with horses.

### 3.2. Welfare as Space

The second discourse refers to the provision of space during air transportation. Although the discourse contributes to establishing horse-friendly transport conditions, it appears to be separate from the first discourse and focuses exclusively on welfare as space. The empirical data clarifies that horses are either transported in air stables, also called jet stalls, or open stalls. Air stables organize the international transport of horses on larger cargo planes. They are made from aluminum and can be firmly anchored on the main deck of the cargo aircraft. A tarpaulin serves as a roof to cover the stable. In contrast, open stalls are composed of partitions that are built around the horses. They are used on planes that exclusively carry horses. The newspaper articles use business-style language to explain the available space inside the stables and borrowed the terms “first class”, “business class”, and “economy or coach class”. A news article published by Horse and Hound in 2019 illustrates the difference between the first and business classes as follows:

“The stalls take up to three horses […] but clients can pay 35% more for “business class” (two horses per stall) or “first class” (single horse, 70% more), for instance for stallions or racehorses running in the Melbourne Cup [52].”

The quotation introduces the three options and mentions that larger stables are used for sport horses or valuable breeding horses. Other news articles and video clips highlight the advantages of business class for sport horses. To ensure that the horses arrive at the peak of their performance, they often have more space on their journey to tournaments, like the Olympic Games. In contrast, the empirical material suggests that smaller horses, broodmares, and leisure horses often travel in threes in a single air stable. An agency representative provided more detailed information about economy-class transportation:

“The biggest thing for the horses is proper space for their size. The guidelines are the horses have to have three inches on either side of them. And what jet stalls can hold are basically 89 inches inside, OK? So, if you put in three horses, they have 28 inches of space. That means your horse theoretically shouldn’t be wider than 22 inches to fit into that space. If they are, then they should go to a half stall, but a half stall is going to cost them usually about 2000 more euros than a third stall. So, there’s a financial thing (agency representative 3).”

Later, the agency representative described the consequences related to space when shipping horses internationally. The limited space could increase stress for horses during transportation. The same representative had noted that if mares do not have enough space to spread their legs inside the stable, they urinate as soon as they leave the air stables. Moreover, less space increases the possibility of horses harassing each other during the flight. For unfamiliar horses, such proximity can be aversive and stressful. Flying grooms react to these problems by applying partition walls or sniffer boards between horses. Lastly, the agency worker concluded that there needs to be more awareness about the consequences of little space because many owners would be willing to pay higher prices for more space.

The earlier academic debate approached space during air transportation in terms of the necessary height to be guaranteed for horses inside the air stables [53]. Similar to the first welfare discourse, the provision of space guarantees equine-appropriate transport conditions. However, this discourse considers horses in terms of their body, not as a species member. Furthermore, the space-as-welfare discourse entails an economic dimension by turning additional space into a purchasable commodity according to human desires. Valuable horses, like competition horses and stallions, often receive more space, regardless of their size. In view of this, horses appear as sentient beings who can suffer and are a manageable resource [54]. While international guidelines set a minimum amount of space inside the air stables to avoid negative welfare [20], the space-as-welfare discourse highlights that the creation of better welfare, the provision of additional space in this case, is a purchasable commodity. Consequently, the discourse implies a shift to positive welfare that is integrated into an economic transaction.

The air stables confine horses to ensure safe and smooth transportation. A standard air stable has the dimensions of 3.17 m (length) × 2.23 m (width) × 2.02 m (height) and provides a width of 75 cm per horse in an economy-class setting. The stable dimensions reduce the movement of horses to keep them calm and immobile, guaranteeing safety during transportation. Inside the air stable, halters secure the horses. In Madness and Civilization [55], Foucault describes a change in confinement practices by identifying how confined spaces have been internally rearranged. Initially, confinement separated the sane and insane. Later, confinement restrained and organized freedom to cure madness by producing acceptable and unacceptable madness [55,56]. Likewise, air and open stables organize the available space within the aircraft to control equine bodies through confinement. The restriction of movement is a disciplinary measure that makes the animals useful to human needs and reduces the resistance to transportation [27]. From this perspective, Streiffer and Killoren [57] introduced the distinction between comparative and agential confinement. Comparative confinement focuses on space by separating areas for animals and humans. In contrast, agential confinement means confining animals for a particular purpose. Confining horses during transportation has two significant purposes. Firstly, it provides safety to protect the horses and the crew on board. Secondly, air stables facilitate smooth transportation of horses for the airport staff and agency workers.

### 3.3. Welfare as the Organization of Transportation

The third welfare discourse targets the organization of air transportation. The discourse comprises knowledge about aviation, meteorology, and transportation logistics to improve horses’ well-being. In detail, welfare practices include optimizing transport routes to avoid unfavorable weather conditions, as well as additional take-off and landing procedures and their modification. A newspaper article summarized the organizational aspects of horse transport as follows:

“The goal, he says, is to make the flight stress-free. The horse-cargo flights sometimes accept long delays or alter course by 1000 miles or more to avoid potential turbulence in the air [58].”

The quotation clarifies that pilots postpone transportation or alter routes to avoid turbulence, even if these actions cause long transportation times. The practice aims to reduce stress for horses because turbulence shakes the aircraft and increases noise. However, the agency workers clarified that transportation times should be as short as possible. Furthermore, the video clips and news articles remarked that pilots tend to avoid sharp descents and ascents. They also fly long curves and use the entire runway for take-off and landing to accelerate and brake gradually. These practices allow the horses inside the air stables to adapt to the change of position of the aircraft without risk of injuries. An article by FreightWaves published in 2019 also mentioned that horse flights get priority for take-off if airplanes are waiting in a queue and the weather conditions are unfavorable. Moreover, the empirical data mentioned that agencies arrange efficient routes to avoid stopovers. An agency representative explained as follows:

“When we talk about welfare, we try to get a direct flight, so the aircraft starts and lands only once because that’s the most critical time. […] except when there is no alternative. For example, when we fly larger numbers of animals in one plane. Calgary is a good example. [There are no direct flights to Calgary]. We try to negotiate a re-routing with the airline for an extra charge. That means the airline flies first to Luxembourg, then to Calgary, and then to Seattle, though the regular route would be Luxembourg, Seattle, and then Calgary (agency representative 1).”

Since direct flights are best for equine welfare, agencies arrange re-routings with airlines to avoid additional stopovers that would include another take-off and landing. The practice targets regular cargo flights that carry horses. Charter flights that transport horses to tournaments and competitions usually fly directly and avoid additional stopovers. Moreover, agencies organize the transport effectively by arranging facilities near the airports or at animal terminals at airports, where the horses spend some time before the flight.

Keywords in this discourse are taken from aviation and logistics, like “cargo plane”, “route”, “turbulence”, “take-off”, and “delay”. The interpretation of these practices from logistics and aviation produces a sense-making equine welfare discourse. In other words, the welfare discourse adds meaning to the practices because they reduce the horses’ stress during transportation. Nevertheless, horses are almost absent from the discourse, while pilots and agency workers appear to be the active decision-makers who maneuver the aircraft and arrange the logistical transport. On the welfare continuum, the discourse depicts these practices to avoid additional suffering of the horses [20]. Equine welfare also concentrates on preventing negative states instead of actively promoting positive welfare [49].

The practices discipline horses to be calm and relaxed during transportation by avoiding unfavorable weather conditions, rough flight maneuvers, and additional take-off and landing procedures. Furthermore, they discipline agency staff and pilots by constituting their professional identity in the equine transportation business. Their expertise relies on knowledge of logistics and aviation. Foucault considered professional identities as changing and shaped through power relations. Concerning his own professional identification, he clarified, “Do not ask me who I am and do not ask me to remain the same” [17]. Agency workers and pilots perform these practices to promote equine welfare. Moreover, the performance constitutes them as professional experts when transporting horses by air. A key characteristic of Foucault’s view of power is that nobody possesses and exercises power. Instead, power “invests” and is “transmitted by” those who exercise it. In other words, power goes through individuals [16]. Human agents involved in transporting horses are produced as professional experts when executing optimal transport conditions for horses.

### 3.4. Welfare as Expressing Individuality

The fourth welfare discourse focuses on the individual personality of horses, including their character traits and preferences, to achieve equine welfare. The newspaper articles and video clips reported on famous sport horses and champions, their choices, and travel habits to address individual welfare needs, and the interviews emphasized taking the unique behavior of horses into account to guarantee optimal welfare during transportation. In different newspapers, the story about the horse American Pharaoh reappeared. The horse travelled several times in the Air Horse One airplane, as follows:

“American Pharoah, who last year became the first Triple Crown winner in 37 years, was perhaps the most high-mileage equine traveler ever, Clark says [that] (trainer) Bob Baffert likes to bring him right back to Santa Anita (a track in the Los Angeles area) after a race. He flew from California to Arkansas, to Kentucky, then back to California, to Maryland for the Preakness, back to California, then to New York for the Belmont, and finally back to California again in the space of three months. That’s probably a record. And it sure didn’t seem to hurt him [59].”

According to the article, American Pharaoh did not seem stressed by air transportation. Other news articles described him as an easy traveler who usually flew with his companion pony, Smokey. Although horses typically remain anonymous, newspaper articles and video clips used the names of famous sport horses like American Pharaoh, Zenyatta, Bella Rose, and Cigar to highlight their individual preferences. The racehorse Zenyatta, for example, always waited a moment before she entered Air Horse One if someone wanted to take a picture. The newspaper articles present famous horses as celebrities who have quirks and needs.

Highlighting the individual needs and preferences of horses entails a welfare dimension. Only recently, animal welfare science has turned to the importance of personality and individuality when analyzing animal well-being [49,60]. The interviewees considered the individual behavior of horses in terms of general flexibility, personal travel experiences, and pairing them with travel buddies. An agency representative explained the following:

“There’s no guarantees [concerning welfare], of course, because horses are animals, and they can […] have a mind of their own at any point in time (agency representative 4).”

The same participant later elaborated the following:

‘We’re able to take a lot of cues from them. But you know, it’s such a case-by-case basis because, for some horses, you have no clues. For some horses, air travel is actually smoother than road transportation. So as far as how smooth the aircraft is. There is not every little bump in the road the horses usually feel. So, I mean, there’s often times the horses will sleep during the flight. When I’ve come back to go give them water, I’ve startled them out of their sleep (agency representative 4).”

The quotations stress that equine welfare during transportation depends on the individual horse. Many decisions about welfare follow case-by-case estimations, relying on the experiential knowledge of flying grooms and agency workers, as well as their understanding of the horses. Furthermore, the agency representative stressed that agency workers get to know the horses to pair them up with their travel buddies in advance. That way, the horses get acquainted with each other. The horses’ characters and experience of air travel influence the pairings. For example, horses who are used to flying are buddied up with horses who are flying for the first time, and nervous horses are paired with calm ones.

Grooms usually get to know the horses in advance to identify transport-related problems like claustrophobia or panic attacks to minimize uncertainty and surprises in the air. Waran et al. [47] noted that flying grooms are professionals with horse skills and expertise in aircraft safety. The core of a groom’s expertise is their ability to quickly connect with unfamiliar horses to understand and interpret their behavior. Though a groom–horse relationship stems from an economic context, interactions rely on recognizing each other as individuals [61]. Flying grooms and horses interpret each other’s actions based on multi-species empathy [62]. In such a view, the practices aim to create positive conditions for horses, emphasizing their integrity. The individual aspects of equine welfare originate in a feeling-based approach to animal welfare, which emphasizes the subjective experience of animals as sentient creatures with emotions and the ability to suffer [20,63]. There is a tendency to consider horses as having an intrinsic value.

Like the previous welfare discourses, the welfare-as-expressing-individuality discourse also produces docile and productive bodies that are politically and economically useful [16,64]. The individual treatment of the horses aims to produce calm horses to facilitate unimpeded transportation. Horses must be docile to adapt to the transportation procedures and the unfamiliar people who handle them [36]. Furthermore, the ability of flying grooms to connect with the animals based on their expertise is in accordance with transportation schedules and procedures to be productive for air transportation.

Resisting transportation leads to further individualized measures. The newspaper articles and agency workers reported that individual horses resist transportation by acting out, disobeying, or having a claustrophobic attack in the air stables. These actions are an expression of the poor well-being of the animals. The flying grooms or agency staff aim to spot horses acting nervously in advance to take measures which calm their behavior. These measures include changing the pairing of horses, providing more space, giving tranquilizers during the flight or, in exceptional cases, stopping the transport of a particular horse. Palmer [27] noted that Foucault developed a power spectrum organizing power by its treatment of resistance. Unstable power relations where resistance is present are located at one end of the spectrum, with pastoral power in the middle, and relationships of domination at the other end. From this perspective, measures like changing the pairing of horses, providing more space, or stopping the transport of particular horses recognize individual resistance. They are located near pastoral power relations, promoting the individual’s well-being. In contrast, using tranquilizers does not leave room for resistance, pointing toward domination.

## 4. Discussion

Equine welfare attempts have founded international organizations like the International Federation for Equestrian Sports [65]. In the field of equine transportation by air, the International Air Transportation Association’s Live Animal Regulations and the Terrestrial Animal Health Code of the World Organization for Animal Health have set guidelines to promote welfare during transportation. Recently, research on horse transportation by air has reevaluated the established transportation practices, intending to develop improved guidelines that go beyond the minimum standards of international legislation. The EFSA Panel on Animal Health and Welfare [13] has stressed that the Live Animal Regulations rely mainly on the experience of the transport business and hence target the equine welfare needs only to a limited extent. The document comprises various sections about container and handling requirements to ensure equine-specific transport conditions, reflecting the first and second welfare discourses identified in the empirical data. This study has illustrated that a critical reassessment of space provided for horses can help implement the existing guidelines. In detail, effective communication between agencies and customers about the space available inside the air stables can facilitate case-by-case decisions for horses to counteract the commodified nature of space within the transport business. Lastly, the welfare-expressing-individuality discourse can help establish equine welfare practices that consider the horses’ agency as co-constitutors of their welfare. While international guidelines establish general welfare criteria, case-by-case decisions considering the horses’ character and history can complement general welfare requirements.

The results of this study inform research in veterinary sciences, biology, and social sciences about different approaches to equine welfare and their impact on humans and horses. Furthermore, the transport industry benefits from this study’s nuanced interpretation of equine welfare practices. Nevertheless, the results may appear biased for an audience unfamiliar with qualitative research methods and Foucauldian discourse analysis because only material published in English and the most popular YouTube clips were used in the analysis.

Lastly, the welfare discourses identified in this study concentrate on carrying sport, leisure, and breeding horses, not on horses transported for slaughter. A significant number of horses being transported for slaughter are carried by air in alarming circumstances. These animals are carried in wooden crates with a stocking density of two to four horses without partitions and without feed or water [66,67]. When transporting horses for slaughter by air, welfare concerns also draw on discourses about space and species-appropriate transport conditions. Nevertheless, welfare differences are rooted in the horses’ treatment as livestock. The juxtaposition of transporting horses for slaughter and sport, leisure, and breeding horses highlights the impact of animal commodification on equine welfare during transportation. In other words, the horses’ economic use and their value as commodities [68] influence their well-being during transportation. Prospective research can target these differences when transporting horses by air, for example, in an ethnographic study where fieldwork enables detailed insight into transport practices.

## 5. Conclusions

This study has identified four equine welfare discourses appearing in the international transportation business of horses. These discourses and their composing practices discipline horses and humans alike. The first welfare discourse focuses on species-specific needs to provide appropriate transport conditions for horses. The provision of feed and water, ventilation, a comfortable temperature, and the separation of horses by sex are existing welfare practices. The second welfare discourse targets space during transportation and is closely linked to the first. Horses are shipped in air stables carrying up to three horses, or in open stables. The third discourse contemplates horse welfare in terms of transport organization and management. Welfare practices include the optimal organization routes and the modification of flight maneuvers and procedures, like long descents and ascents, and prolonged braking and acceleration periods. Lastly, the fourth discourse highlights individuality as necessary for equine welfare by considering each horse’s unique character and experiences. Welfare practices comprise reading and responding to horse behavior and pairing horses with travel buddies.

The different welfare discourses complement and oppose each other, but often appear in clusters. Despite their different practices, orientations, and underlying welfare views, they construct horses and humans similarly. On the one hand, all discourses produce active professional experts responsible for equines welfare during transportation. Nevertheless, their expertise differs between experts in equine needs, horse interaction and communication, and experts in aviation and logistics. Performing different welfare practices shapes their professional identities. On the other hand, all discourses, regardless of their view, consider horses as sentient beings with the ability to suffer. The welfare practices mainly avoid discomfort and produce calm and inactive horses that endure the transport procedures and, ideally, rest or sleep during the flight.

## Data Availability

The interviews presented in this study are available on request from the corresponding author due to data protection measures to protect the interviews privacy. The newspaper articles and YouTube clips presented in the study will be made available by the author on request. Informed consent was obtained from all subjects involved in the study.
